# Surgical Management of Metastatic Breast Cancer: A Mini Review

**DOI:** 10.3389/fonc.2022.910544

**Published:** 2022-05-04

**Authors:** Takayuki Ueno

**Affiliations:** Cancer Institute Hospital, Japanese Foundation for Cancer Research, Tokyo, Japan

**Keywords:** metastatic breast cancer, oligometastasis, locoregional therapy, surgical management, *de novo* stage IV

## Abstract

Breast cancer with distant metastases is a systemic disease. While systemic therapies are the main treatment strategy, locoregional therapy for metastatic breast cancer (MBC) is generally palliative only. However, recent progress in systemic and local therapies has improved the prognosis of patients with MBC and some may expect long-term survival. More vigorous local therapies for MBC may, therefore, be clinically justified in selected patients. A number of clinical trials and studies have investigated the clinical significance of surgical therapy for primary tumors and distant metastases in patients with MBC. Four prospective randomized trials and multiple retrospective studies have investigated the benefit of surgical resection of primary lesions in patients with MBC, with conflicting results. There have been a number of case-control studies examining the impact of surgical resection of distant metastases, but the benefit of this approach in terms of survival is controversial because selection bias is unavoidable in retrospective studies. The present review discusses the state of the literature relating to local management of the primary breast cancer through surgical resection and surgical management of distant metastatic lesions including pulmonary and liver metastases with future perspectives.

## Introduction

Distant metastasis of primary breast cancer is a systemic disease. The treatment for metastatic breast cancer (MBC) mainly comprises systemic therapies such as endocrine therapy, chemotherapy, and targeted therapies with the primary intention of palliation. Curative treatment is not attempted in most cases. However, MBC can vary widely in terms of tumor burden, affected organs, cancer subtype, and systemic condition (1, 2), and the overall survival varies from a few months to decades. Advances in systemic therapies, including the development of targeted therapies and immune-checkpoint inhibitors, have improved the prognosis of MBC, with long-term survival achieved in some cases (3). Thus, local therapies for MBC may prolong survival rather than provide palliative therapy in selected patients, justifying the use of more vigorous local therapies. There have been a number of clinical trials and studies investigating the clinical significance of surgical therapies for survival among patients with MBC. The present review provides a comprehensive overview of surgical approaches for both local management of the primary breast cancer and for distant metastatic lesions including lung and liver metastases.

## Surgery for the Primary Breast Cancer in Patients With *De Novo* MBC

Local and systemic therapies are applied to control the primary breast cancer in patients with MBC. Local therapies are considered subsidiary to systemic therapies in most cases, as long as the latter are effective. Resection of the primary breast cancer is mostly palliative in patients with MBC, for purposes such as controlling bleeding and ulceration, minimizing infection and pain, and to address impaired wound healing. A large number of retrospective studies have examined the impact of locoregional therapies including surgery on survival in patients with MBC, and several meta-analyses have subsequently been conducted. One meta-analysis of 19 retrospective studies revealed the pooled hazard ratio (HR) of local surgery vs. systemic therapy alone to be 0.65 with a 95% confidence interval (95% CI) of 0.60–0.71 (p < 0.01), indicating that overall survival was significantly improved by surgical resection of the primary lesion (4). This finding was supported by another meta-analysis of 12 retrospective studies (odds ratio, 0.65; 95% CI, 0.59–0.72) (5). A large meta-analysis of 34 retrospective studies and 3 prospective randomized studies found surgical resection to significantly reduce mortality (HR, 0.64; 95% CI, 0.60–0.68) (6). Although surgical resection of the primary tumor appears to be the optimal approach according to these meta-analyses, the use of retrospective studies alone risks introducing selection bias.

There have been four prospective randomized trials conducted to investigate whether resection of the primary breast lesions can improve survival (**Table 1**). An Indian trial involving 350 patients randomly assigned participants to locoregional treatment or no locoregional treatment (7). Prior to surgery, patients with resectable hormone-sensitive breast cancer received endocrine therapy until disease progression was observed, while those with unresectable breast tumors received chemotherapy. Patients with partial or complete responses were randomized. Over a median follow-up time of 23 months, the overall survival was not significantly different between locoregional and no locoregional treatment groups (median: 19.2 and 20.5 months, respectively; p = 0.79; **Table 1**).

**Table 1:** Surgery for primary site.

	Region	Period	Inclusion	No. of registered patients	Criteria for randomization	No. of randomized patients	systemic therapy before surgery	Primary Endpoint	Secondary Endpoint	Median follow-up (months)	Surgery in the surgery arm	Median overall survival (months)				Locoregional progression				QOL	Reference
												Surgery	No surgery	HR [95%CI]	P value	Surgery	No surgery	HR [95%CI]	P value		
India	India	2005-2013	resectable hormone sensitive primary breast tumor or unresectable primary tumors with complete or partial response to anthracycline-containing chemotherapy	440		350	endocrine therapy or anthracycline-based or sequential or concurrent anthracycline taxane regimen	Overall survival	Locoregional progression-free survival Distant progression-free survival Health-related quality of life	23	Modified radical mastectomy 72% Breast conserving surgery 23%	19.2	20.5	1.04 [0.81-1.34]	0.79	(median locoregional progression-free survival				Not reported	(7)
																not attained	18.2 months	0.16 [0.10-0.26]	<0.0001		
											All patients with surgery received axillary dissection.										
MF07-01	Turkey	2007-2012	De novo stage IV breast cancer Primary breast tumor amenable for complete resection	278		278	None	Overall survival	Rates of locoregional progression/relapse 30 day-mortality	40	Mastectomy 74% Breast conserving surgery 26%	46	37	0.66 [0.49-0.88]	0.005	1%	11%		0.001	Not reported	(8, 9)
											Axillary dissection 92.8% Sentinel lymph node biopsy 17% (axillay dissection with positive node)										
ABCSG28 POSYTIVE trial	Austria	2011-2015	De novo metastatic disease. Operable breast cancer at primary	90		90	None	Overall survival	Time to distant progression. Time to locoregional progression Quality of life assessment	37.5	Mastectomy 71% Breast conserving surgery 29%	34.6	54.8	0.691 [0.358-1.333]	0.267	8.9%	17.8%		0.2148	EORTC QLQ-C30 & QLQ BR23 no difference	(10, 11)
											Axillary dissection 93% Sentinel lymph node biopsy only 7%										
ECOG-ACRIN E2108	USA	2011-2015	De novo stage IV breast cancer	390	No progression of distant disease following 4-8 months of optimal systemic therapy	256	4-8 months of optimal systemic therapy based on patient and disease features	Overall survival	Time to locoregional progression Health-related quality of life Absolute value of circulating tumor cell burden Collection of biological samples	53	Surgery in the locoregional therapy arm: 85.6%	54.9	53.1	1.11 [0.82-1.52] (90%CI)	0.57	16.3%	39.8%	0.34 [0.21-0.56]	<0.001	FACT-B TOI lower in the surgery group at 18 months (p = 0.01) not different at registration, randomization, 6, 30 months	(12)
											Free surgical margin: 91.6%										

In the MF07-01 trial, which was conducted in Turkey, 278 patients were randomly assigned to either locoregional treatment followed by systemic treatment or systemic treatment without locoregional treatment. Over a median follow-up time of 40 months, overall survival was significantly improved by locoregional treatment compared with systemic treatment alone (46 and 37 months, respectively; p = 0.005; **Table 1**) (8, 9). However, the two groups in the MF07-01 trial were not well-balanced in terms of subtype as the locoregional treatment group contained more participants with hormone-receptor-positive cancer and less with triple-negative cancer compared with the no locoregional treatment group (85.5% vs. 71.8%, p = 0.01 and 7.3% vs. 17.4%, p = 0.01, respectively). Thus, the results should be interpreted with caution.

The Austrian Breast and Colorectal Cancer Study Group 28 (ABCSG28) POSYTIVE trial was conducted in Austria and originally planned 254 participants but was stopped prematurely due to poor recruitment. In this study, 90 previously untreated patients with stage IV breast cancer were randomly assigned to either surgical resection of the primary tumor followed by systemic therapy or primary systemic therapy (10), with resulting survival rates of 34.6 and 54.8 months, respectively, over a median follow-up period of 37.5 months (p = 0.267). Surgery was also not found to significantly improve quality of life (QOL) as measured by European Organisation for Research and Treatment of Cancer (EORTC) QLQ-C30 and QLQ BR23 (11).

Two meta-analyses of these three prospective studies revealed that surgical treatment of primary lesions does not significantly prolong overall survival (HR, 0.81; 95% CI, 0.57–1.14 in the study by Gera et al. and odds ratio, 0.81; 95% CI, 0.60–1.11 in the study by Tsukioki et al.) (5, 6).

The Eastern Cooperative Oncology Group (ECOG) E2108 trial conducted in the USA enrolled 256 patients with *de novo* stage IV breast cancer who showed no progression following 4–8 months of optimal systemic therapy. Participants were randomly assigned to early local therapy or continued systemic therapy, with no significant difference in survival seen over a median follow-up time of 53 months (54.9 vs. 53.1 months, respectively; p = 0.57) (12). Exploratory *post-hoc* subgroup analyses revealed that survival was reduced in the local therapy arm of 20 participants with triple-negative breast cancer (HR, 3.33; 95% CI, 1.09–10.12) but similar among those with other subtypes (HR, 1.05; 95% CI, 0.49–2.24 for human epidermal growth factor receptor 2 [HER2]-positive; HR, 0.88; 95% CI, 0.56–1.39 for hormone-receptor-positive HER2-negative). Thus, the impact of early local therapy differs according to subtype, although conclusions should be made with caution because of the small size of each subgroup. The QOL measured by the Functional Assessment of Cancer Therapy - Breast Trial Outcome Index (FACT-B TOI) was lower at 18 months postrandomization for participants undergoing early local therapy (p = 0.01), which was not different at any other time point (12).

A meta-analysis of the four aforementioned prospective randomized trials (using the results of the E2108 trial reported in the 2020 American Society of Clinical Oncology Annual Meeting (13) because the trial had not been published yet) revealed that locoregional therapy does not improve overall survival among the intention-to-treat population (HR, 0.97; 95% CI, 072–1.29) (14). Subgroup analyses showed that locoregional therapy did not improve overall survival for any subtype, including triple-negative breast cancer (HR, 1.4; 95%CI, 0.50–3.91), hormone-receptor-positive cancer (HR, 0.96; 95%CI, 0.65–1.43) and HER2-positive cancer (HR, 0.93; 95%CI, 0.68–1.28).

Although one prospective trial, the MF07-01 trial, in which the randomized arms were not well-balanced, showed survival benefit, the results of the prospective trials and the meta-analysis indicate that surgical treatment of primary lesions may not improve survival in patients with MBC. However, the impact of surgical therapy on survival for different breast cancer subtypes and different sites of metastases has not been widely researched and warrants further investigation in future trials. A recent multicenter prospective registry study (the BOMET MF14-01 trial) found that primary surgery significantly prolonged survival for patients with *de novo* stage IV breast cancer with bone metastasis only after adjustment by multivariate analysis (median follow-up time: 3 years; HR, 0.41; 95%CI, 0.30–0.57) (15). Although this was not a randomized trial, a prospective registry approach is useful and the results are promising. A multicenter phase III trial is currently ongoing in Japan, investigating the benefit of primary tumor resection in terms of survival of patients with stage IV breast cancer who are not refractory to systemic therapy (16).

## Surgery for Metastatic Lesions in Patients With MBC

Distant metastasis is considered evidence of systemic spread of breast cancer; therefore, surgical management of metastatic lesions is not the standard of care. However, the concept of “oligometastases” was introduced by Hellman et al. and has changed the view of metastatic disease within the field (17). Oligometastases refer to tumors early in the chain of progression, when metastases are limited in number and location, while micrometastases are small in size but extensive in number (17). The concept of an oligometastatic state suggests that, in some cases, metastases could be treated with curative intent using strategies involving local and systemic treatment. Indeed, long-term survival has been achieved in some patients who underwent surgery for distant metastatic lesions (18). The median overall survival after resection of pulmonary metastases has been reported to be 96.9 months, which is longer than generally expected for distant MBC (19). Although the definition of oligometastasis varies (20–23), the following have been suggested: limited number and size of metastatic lesions, potentially amenable for local treatment, and potential for achieving complete remission, which were suggested by the International Consensus Guidelines for Advanced Breast Cancer (24). Metastasis-directed therapies include surgery and radiation therapy such as stereotactic radiotherapy and external-beam radiation therapy (EBRT). The benefits of stereotactic ablative radiotherapy (SABR) as a local therapy for distant metastases (mainly lung and bone metastases) have only been reported in one randomized trial involving patients with different types of cancers (25). Over a median follow-up period of 51 months, the 5-year overall survival rate was 42.3% in the SABR arm and 17.7% in the standard of care arm (p = 0.006).

This review focuses on surgical resection of distant metastases but there have been no reported randomized studies investigating surgical resection of metastatic lesions to date. Because surgical approach requires different considerations depending on the location of metastases, surgery for distant metastatic lesions is discussed according to metastatic sites. In patients with brain metastases, local therapies including surgical resection, stereotactic radiotherapy, or whole-brain radiotherapy are the mainstay of treatment while recent progress in systemic therapy as well as local therapy has made the approach more individualized, especially for HER2-positive breast cancer (24). Surgical resection of metastatic sites other than brain, lung and liver for therapeutic purposes has been reported in few studies and, in this review, surgery for pulmonary and liver metastases is further discussed.

## Surgery for Pulmonary Metastases

Resection of pulmonary metastasis is performed not only for therapeutic purposes but to obtain diagnostic biopsies. Recently, this approach has become an important method for determining the hormone-receptor and HER2 status of metastatic lesions because of the conversion of subtypes between primary and metastatic cancers (26–28). Numerous retrospective studies examining the clinical significance of resection of pulmonary metastasis originating from breast cancer have reported median overall survival rates ranging from 20 to 96.9 months (19, 29–31). A large meta-analysis involving 1937 patients from 16 studies found the pooled 5-year survival rate to be 46% (32), and adverse prognostic indicators included a short disease-free interval (< 3 years), incomplete resection of metastases, multiple metastases, and hormone-receptor-negative status of metastatic tumors. However, because these studies are retrospective and selection bias for resection of metastasis is unavoidable, the impact of resection of pulmonary metastases on survival remains unclear. Some case-control studies have demonstrated the positive impact of resection of pulmonary metastases on survival (**Table 2**). A study by Staren et al. compared 33 patients who were treated primarily with surgical resection of pulmonary metastases with 30 patients who were treated primarily with systemic chemoendocrine therapy (33). Twenty of 33 patients in the surgery group received pulmonary resection alone. The mean survival in the surgery group was 55 months, which was significantly longer than 33 months in the systemic therapy group (p = 0.023). Improved survival was observed even when patients with single pulmonary lesions were compared (58 months vs. 34 months, p = 0.025). The 5-year survival rate was significantly better in the surgery group compared with the systemic therapy group (36% and 11%, respectively, p = 0.017), even though the majority of patients who underwent surgery (20 out of 33) did not receive systemic therapy. However, this may suggest that patients who underwent surgery had good general conditions and good prognosis at the time that treatment decisions were made.

**Table 2:** Resection of metastatic lesions.

		Year of publication	No. of patients		No. of patients by metastasis type			Median Survival (months)					5-year overall survival			Reference publication
			surgery	no surgery		surgery	no surgery	surgery	no surgery		P value		surgery	no surgery	P value	
**Pulmonary metastasis**																
Staren ED et al.		1992	33	30	single	27	20	(Mean survival)					36%	11%	0.017	(33)
					multiple	6	10	55		33		0.023				
Yhim HY et al.		2010	15	30	single	11	10	not attained		34.3		0.011	(4-year overall survival)			(34)
					multiple	4	20						82.1%	31.6%	0.001	
**Liver metastasis**																
Mariani P et al.		2013	51	51	single	36	Not reported	Not reported		Not reported		Not reported	(3-year overall survival)			(35)
					multiple	15	Not reported						80.7%	50.9%	<0.0001	
Sadot E et al.		2016	69	98	solitary	44	29	50		45		0.5	38%	39%	0.98	(36)
					>5	7	32									
Abbas H et al.		2017	23	27	solitary	15	3	49		20		0.001	56%	25%	Not reported	(37)
					>5	0	17									
Ruiz A et al.		2018	139	523				74		13		<0.001	57%	10%	Not reported	(38)
			Propensity Score Matching													
			49	49	single	19	20	82		31		<0.001	69%	24%	<0.001	
					multiple	30	29									

A Korean study comparing 15 patients who underwent pulmonary surgery followed by systemic therapies with 30 patients who received systemic therapy alone found that, over a median follow-up time of 50.1 months, the median overall survival was not reached in the surgery group and was 34.3 months in the no surgery group (p = 0.011) (34). The 4-year overall survival was significantly better in the surgery group compared with no surgery (82.1% vs. 31.6%, respectively; p = 0.001). Multivariate analysis for overall survival revealed short disease-free interval (<24 months) and breast cancer subtype (HER2 and triple-negative) to be independent unfavorable prognostic factors while pulmonary surgery did not remain independent.

Resection of pulmonary metastases may prolong survival of some patients with pulmonary metastases, potentially because patients who are suitable for surgical treatment are likely to have favorable general conditions and disease status. This may explain why surgical resection of pulmonary metastases was not an independent factor in the multivariate analysis of the Korean study (34) and resection is currently only considered for selected patients. However, systemic therapies and surgical techniques including thoracoscopic surgeries have improved in recent years, meaning that the range of patients who are potential candidates for curative treatment is increasing.

## Surgery for Liver Metastases

There has been research into improving the survival of patients with liver metastases from breast cancer, including the application of hepatectomy and radiofrequency ablation as well as systemic therapies (39). A number of retrospective studies have reported the safety and benefits of resection of liver metastases, with median overall survival ranging from 27 to 74 months (40–43). A large systematic review including 19 studies reported the median overall and 5-year survival to be 40 months and 40%, respectively, among 553 patients with breast cancer liver metastases who underwent hepatectomy, although positive surgical margin of liver and hormone refractory were found to be adverse prognostic factors (44). These retrospective analyses, however, cannot identify the specific benefit of resection of liver metastases because the impact of surgical procedure on survival is not known.

Several case-control studies have compared surgical and non-surgical approaches for the treatment of breast cancer with liver metastases (**Table 2**). One of these, conducted in France, showed surgical resection to significantly improve the 3-year overall survival (80.7% vs. 50.9% in the no surgery group p < 0.0001) (35). This study included participants with bone metastases other than liver metastases but not other sites of distant metastases. Multivariate analysis revealed surgical resection of liver metastases and absence of bone metastases to be independent favorable prognostic factors. In contrast, a study from the Memorial Sloan Kettering Cancer Center did not find surgery to have a significant benefit in terms of survival (median follow-up time of 73 months, median overall survival 50 months compared with 45 months in the no surgery group, p = 0.5) (36). The 5-year overall survival rates were 38% and 39% for the surgery and no surgery groups, respectively (p = 0.98). A UK study found that 23 patients who underwent surgical resection had improved median overall survival compared with 27 patients who underwent chemotherapy (49 vs. 20 months, p < 0.001) (37). However, the sample size of this study was small, and so interpretations should be made with caution. A European case-matched comparative study found the median overall survival among 139 patients who underwent surgery to be 74 months, compared with 13 months among 523 patients who did not (p < 0.001) (38). After propensity-score matching to balance the groups, 49 patients remained per group, with median overall survivals of 82 and 31 months and 5-year overall survival rates of 69% and 24% in the surgery and no surgery groups, respectively (p < 0.001). The number of patients left after matching was small and so conclusions should still be considered with caution. Furthermore, this may indicate that large differences existed in patient baseline characteristics between groups.

A meta-analysis of the three studies described above (36–38) was carried out in order to determine management guidelines for liver metastases from extrahepatic primary cancers (45). This found no improvement in survival from surgical treatment of liver metastases (odds ratio: 0.29; 95% CI, 0.07–1.26) and concluded that hepatectomy for breast cancer liver metastases is not recommendable and can only be justified in selected patients with favorable prognoses and general conditions (45).

The evidence that is currently available does not demonstrate a clear survival benefit of hepatectomy for patients with breast cancer with liver metastases. Thus, hepatectomy should be considered only in carefully selected patients who have favorable systemic and disease conditions.

## Discussion

The recent progress in systemic therapies and local management has contributed to the prolonged survival that can be expected for patients with MBC (3). However, the benefit of surgical therapies of primary tumors or distant metastases remains unclear. While curative therapy may be possible for some patients with MBC, the specific details of which patients and how treatment can be applied are yet to be defined. Randomized studies are optimal but recruitment for such studies takes many years and huge efforts, complicating the interpretation because systemic therapies will have progressed by the time the results can be published. It is critically important to accumulate real-world data, and analysis using not just conventional bioinformatics approach but advanced approach including artificial intelligence will improve the identification of suitable candidates for vigorous local treatment. Scientific approaches including detection of minimal-residual disease using circulating tumor DNA and molecular imaging, as well as defining the difference between oligometastatic and polymetastatic states using microRNA and other molecular approaches, will help to select patients and improve the current understanding of the oligometastatic state (46–50). This will enable the development of treatment strategies for patients with MBC with the aim of curing the disease.

## Author Contributions

TU contributed to the study design, conception, literature search, review, drafting and editing the manuscript. TU contributed to the article and approved the submitted version.

## Conflict of Interest 

The author declares that the research was conducted in the absence of any commercial or financial relationships that could be construed as a potential conflict of interest.

## Publisher’s Note

All claims expressed in this article are solely those of the authors and do not necessarily represent those of their affiliated organizations, or those of the publisher, the editors and the reviewers. Any product that may be evaluated in this article, or claim that may be made by its manufacturer, is not guaranteed or endorsed by the publisher.
